# Evolution of systemic therapy for advanced HCC patients: Did we make progress in 2022?

**DOI:** 10.1097/HC9.0000000000000117

**Published:** 2023-05-15

**Authors:** Jeffrey Sum Lung Wong, Roland Leung, Tan To Cheung, Thomas Yau

**Affiliations:** 1 Department of Medicine, Queen Mary Hospital, The University of Hong Kong, Hong Kong, China; 2 Department of Surgery, Queen Mary Hospital, The University of Hong Kong, Hong Kong, China

Great strides have been made in the systemic treatment of advanced HCC in the last 6 years. Approved tyrosine kinase inhibitors (TKIs) have been expanded to include lenvatinib (LEN), regorafenib, and cabozantinib (CABO). Meanwhile, immune checkpoint inhibitors (ICI) including nivolumab (NIVO) ± ipilimumab, pembrolizumab (PEMBRO), and atezolizumab (+ bevacizumab) (ATEZO+BEV) have revolutionized advanced HCC treatment. The past year saw the publication of important clinical trials, all of which evaluated ICIs but differed in ICI partners. They can be broadly categorized into single agents ICI, dual-ICIs, and ICI + antiangiogenic agent studies (Table 1).

## SINGLE-AGENT ICIS

In the first-line setting (1 L), 2 phase 3 trials comparing anti-PD1 to sorafenib (SOR) released updates. The final analysis of CheckMate-459, which compared NIVO to SOR in a superiority design, reported a median overall survival (mOS) of 16.4 months for NIVO and 14.7 months for SOR (HR: 0.85, *p* = 0.075).^1^ The objective response rate (ORR) was 15% and 7%, respectively. Grade 3 or 4 treatment-related adverse events (TRAEs) occurred in 18% and 4% of NIVO, compared with 47% and 2% of SOR. Though overall survival (OS) improvement was not statistically significant with NIVO, there was a sustained separation of Kaplan-Meier curves and numerically higher OS at 12-, 18-, and 24-month landmarks. In addition, 20% of SOR patients received ICIs later, which potentially introduced confounding effects on the OS results. The second study, RATIONALE-301, compared tisleilizumab to SOR in a noninferiority design.^2^ mOS was 15.9 months for tisleilizumab and 14.1 months for SOR (HR: 0.85), meeting the cutoff for noninferiority but not superiority. ORR was 14.3% versus 5.4%, and grade ≥3 TRAE occurred in 22.2% and 53.4% of tisleilizumab and SOR arms, respectively. The results from these two studies are quite consistent, with NIVO and tisleilizumab demonstrating similar mOS and ORR, while both showed favorable safety profiles over SOR. Taken together, they suggest that single-agent ICI might be considered as an alternative to TKIs, or in patients for whom antiangiogenics carry substantial risks.

In the second-line setting (2 L), KEYNOTE-394, evaluating PEMBRO versus placebo in Asian patients previously treated with SOR, was published.^3^ Notably, most patients were Chinese (85%) and HBV carriers (79%). mOS was significantly longer with PEMBRO (14.6 vs. 13.0 months, HR: 0.79, *p* = 0.018), with progression free survival (PFS) and ORR also favoring PEMBRO. These results are interesting as KEYNOTE-240, which was identically designed except with international enrollment, did not show statistically significant OS benefit for PEMBRO. Though one potential explanation of this difference is that most KEYNOTE-240 patients were non-Asian and not predominately HBV infected, it should be noted that the HR for OS (0.79 and 0.781) and PFS (0.74 and 0.775) were similar between the two studies.

## DUAL-ICIS

Combined anti-CTLA-4 and anti-PD-1 ICIs have been approved in 2 L based on CheckMate-040 cohort 4 (NIVO + ipilimumab ). In 1 L, the HIMALAYA trial has, for the first time, demonstrated superior outcomes of dual-ICI compared with TKIs.^4^ In HIMALAYA, mOS was 16.4 months with the single tremelimumab regular interval durvalumab regimen (STRIDE), 16.6 months with single-agent durvalumab and 13.8 months with SOR, with STRIDE demonstrating statistically significant benefit over SOR (HR: 0.78, *p* = 0.0035) and durvalumab showing noninferiority (HR: 0.86). However, median PFS (mPFS) was not significantly different versus SOR (HR for STRIDE: 0.9, for durvalumab: 1.02). Grade 3–4 treatment-emergent adverse events occurred in 50.5% with STRIDE, 37.1% with durvalumab, and 52.4% with SOR. Based on these results, the US Food and Drug Administration has approved the STRIDE regimen for use in 1 L advanced HCC.

## COMBINED ANTI-PD-1/L1 ICIS AND ANTIANGIOGENIC AGENTS

IMBrave150 established the new treatment standard in 1 L advanced HCC and demonstrated the potential synergy between ICIs and antiangiogenic agents, in this case, anti-VEGF-mAbs. Updated follow-up data were recently published, with mOS reaching 19.2 months for ATEZO-BEV and 13.4 months for SOR (HR: 0.66, *p* < 0.001).^5^ mPFS was 6.9 versus 4.3 months (HR: 0.65, *p* < 0.001); and grade 3–4 TRAEs occurred in 43% and 46% of patients, respectively. The results are broadly in line with those of the primary analysis.

A wealth of phase 3 data on another approach of combining ICIs with antiangiogenic, this time multitargeted TKIs, was released recently. COSMIC-312 compared ATEZO + CABO to SOR in 1 L.^6^ mPFS was 6.8 months versus 4.2 months (HR: 0.63, *p* = 0.0012) significantly favoring ATEZO + CABO, but mOS was negative (15.4 vs. 15.5 months, HR: 0.9, *p* = 0.44). Grade 3 and 4 TRAEs occurred in 51% and 3% of ATEZO + CABO, compared with 30% and 2% of SOR. Based on these results, the authors concluded that additional studies are needed for ATEZO + CABO to be established in advanced HCC.

In addition, results of LEAP-002 comparing PEMBRO + LEN to LEN + placebo, as well as the NCT03764293 trial comparing camrelizumab + rivoceranib (CAM + RIVO) to SOR, both for 1 L, were recently announced.^7^,^8^ In LEAP-002, mOS was 21.2 months for PEMBRO + LEN and 19.0 months for LEN + Placebo (HR: 0.84), whereas mPFS was 8.2 months and 8.0 months, respectively; neither result reached the superiority thresholds. Grade 3–4 TRAEs occurred in 61.5% and 56.7% of patients respectively. Finally, in the CAM + RIVO trial, mOS was 22.1 months for CAM + RIVO and 15.2 months for SOR, whereas mPFS was 5.6 months and 3.7 months, respectively, both significantly favoring CAM + RIVO. Grade 3–4 TRAEs occurred in 80.5% and 52% of CAM-RIVO and SOR patients, respectively.

Overall, 2 observations can be made from these results. Firstly, current ICI-TKI combinations seem to have additional toxicity without efficacy benefit over ATEZO-BEV. Both COSMIC-312 and LEAP-002 failed to demonstrate OS benefit over their TKI control arms. Although the CAM + RIVO trial was positive, a considerable number of patients, especially in the SOR arm (18.8%), withdrew consent. In addition, most patients were Asian (82.7%) and HBV infected (74.5%), with the efficacy data of CAM + RIVO not obviously superior compared with the IMBrave150 Chinese cohort, which has comparable demographics.^9^ Meanwhile, higher incidences of grade ≥3 TRAEs were observed for all 3 studies, both compared with their control arms and to ATEZO-BEV in IMBrave150. Secondly, single-agent TKI, especially LEN, remains a viable option in 1 L. The OS of control-arm TKIs has improved continuously since their registration trials, most remarkably reaching a mOS of 19.0 months for LEN + placebo in LEAP-002, vastly exceeding that of LEN in the REFLECT trial (13.6 mo).

## DUAL-ICIS AND ANTIANGIOGENIC AGENTS

Combining dual-ICIs and antiangiogenic agents has so far remained an investigational approach. The first such prospective trial was recently published: in the phase 1/2 CheckMate-040 cohort 6 study, NIVO + ipilimumab + CABO demonstrated a mOS of 22.1 months, mPFS of 4.3 months, and ORR of 29% in 1 L.^10^ The incidence of grade 3–4 TRAEs was 74%. Of note, the median duration of response was still not reached after 32.0 months of follow-up. Further trials in this direction, including the recently opened phase 1/2 RELATIVITY-106 investigating the combination of NIVO + BEV with the anti-LAG-3 ICI relatlimab in 1 L, are needed to determine the added value of this approach.

In conclusion, 2022 was another fruitful year for advanced HCC therapeutics, with new treatment options being approved and ever more data published for greater treatment precision and novel drug development directions. We took another step forward toward defeating this deadly and ever-more-common condition (Table 1).

**TABLE 1:** List of advanced-HCC trial results released in 2022

	Trials	Phase	Line	Experimental arm	Control arm	mOS (mo)	median PFS (mo)	ORR (%)	Grade 3–4 AEs (%)
Single-agent ICI	CheckMate-459	3	1	NIVO	SOR	16.4 vs. 14.7	3.7 vs. 3.8	15 vs. 7	22 vs. 49
	RATIONALE-301	3	1	TIS	SOR	15.9 vs. 14.1	2.2 vs. 3.6	14.3 vs. 5.4	22.2 vs. 53.4
	KEYNOTE-394	3	2	PEMBRO	Placebo	14.6 vs. 13.0^a^	2.6 vs. 2.3^a^	12.7 vs. 1.3	13.3 vs. 5.9
Dual-ICIs	HIMAYALA	3	1	STRIDE	SOR	16.4 vs. 13.8^a^	3.78 vs. 4.07	20.1 vs. 5.1	50.5 vs. 52.4
ICI + antiangiogenic agents	IMBrave150	3	1	ATEZO + BEV	SOR	19.2 vs. 13.4^a^	6.9 vs. 4.3^a^	30 vs. 11	43 vs. 46
	COSMIC-312	3	1	ATEZO + CABO	SOR	15.4 vs. 15.5	6.8 vs. 4.2^a^	13 vs. 5	54 vs. 32
	NCT03764293	3	1	CAM + RIVO	SOR	22.1 vs. 15.2^a^	5.6 vs. 3.7^a^	25.4 vs. 5.9	80.5 vs. 52
	LEAP-002	3	1	PEMBRO + LEN	LEN + placebo	21.2 vs. 19.0	8.2 vs. 8.0	26.1 vs. 17.5	61.5 vs. 56.7
	CheckMate-040 cohort 6	1/2	1	NIVO + IPI + CABO	—	22.1	4.3	29	74

^a^ Statistically significant results.

Abbreviations: AEs, adverse events; ATEZO, atezolizumab; BEV, bevacizumab; CABO, cabozantinib; CAM, camrelizumab; ICI, immune checkpoint inhibitor; IPI, ipilimumab; LEN, lenvatinib; mOS, median overall survival; NIVO, nivolumab; ORR, objective response rate; PEMBRO, pembrolizumab; RIVO, rivoceranib; SOR, sorafenib; STRIDE, single tremelimumab regular interval durvalumab; TIS, tisleilizumab.
